# Release and Disintegration Properties of Poly(lactic Acid) Films with Allyl Isothiocyanate-β-Cyclodextrin Inclusion Complexes for Active Food Packaging

**DOI:** 10.3390/molecules29245859

**Published:** 2024-12-12

**Authors:** Cristina Muñoz-Shugulí, Francisco Rodríguez-Mercado, Abel Guarda, María José Galotto, Alfonso Jiménez, María Carmen Garrigós, Marina Ramos

**Affiliations:** 1 Facultad de Ciencias, Escuela Superior Politécnica de Chimborazo (ESPOCH), Riobamba EC060155, Ecuador; cristina.munoz@espoch.edu.ec; 2 Packaging Innovation Center (LABEN-Chile), University of Santiago of Chile (USACH), Santiago 9170124, Chile; francisco.rodriguez.m@usach.cl (F.R.-M.); abel.guarda@usach.cl (A.G.); maria.galotto@usach.cl (M.J.G.); 3 Department of Analytical Chemistry, Nutrition & Food Sciences, University of Alicante, 03690 Alicante, Spain; alfjimenez@ua.es (A.J.); mc.garrigos@ua.es (M.C.G.)

**Keywords:** PLA, allyl isothiocyanate, β-cyclodextrin, active food packaging, disintegration, release

## Abstract

This study aimed to enhance the properties and compostability of active poly(lactic acid) (PLA) films by incorporating β-cyclodextrin (β-CD) inclusion complexes with allyl isothiocyanate (AITC). Films were prepared using melt extrusion and characterized based on their structural, chemical, morphological, thermal, and barrier properties. These inclusion complexes improved the thermal stability and moisture absorption of films, enhancing disintegration under composting conditions. The release of AITC in the vapor phase was responsive to relative humidity, maintaining the antimicrobial functionality at low values and releasing effectively at higher humidity levels, with a maximum release at 100%. Incorporating 5% and 10% β-CD:AITC complexes accelerated disintegration under composting conditions, reducing the time by 5 days for disintegration compared to pure PLA, achieving up to 90% in 23 days. These results, with a general improvement in functional properties, suggest that PLA films with β-CD:AITC are promising for developing sustainable, biodegradable antimicrobial packaging solutions for food applications.

## 1. Introduction

Active packaging has gained significant interest in the context of increasing demands for food safety and quality as well as environmental concerns related to traditional plastic packaging. Unlike traditional methods that directly add active ingredients, such as antioxidants and antimicrobials, to food, active packaging strategies are based on the incorporation of these substances into the packaging material. This method extends the effectiveness of the active ingredients within the packaging system and addresses sustainability concerns [1]. Despite these advancements, achieving effective controlled release of active additives from the packaging materials remains challenging, highlighting the growing interest in microencapsulation technologies [2]. In addition, shifting toward green packaging solutions has become a priority, sparking intense research into biodegradable polymer materials [3,4].

Microencapsulation typically involves enveloping the potentially volatile active chemicals in a continuous film of tiny droplets or particles, using natural or synthetic polymers as the encapsulating material. These particles, known as microcapsules, can significantly enhance active compounds’ stability, solubility, and release dynamics [5]. Cyclodextrins have proven to be adequate encapsulating materials due to their globular structure, which allows them to host specific molecules through the formation of covalent bonds [6,7]. Among them, β-cyclodextrin (β-CD) is particularly notable due to its moderate molecular cavity and cost-effective production. β-CD microencapsulation shows a promising strategy for enhancing different compounds’ stability, solubility, and release characteristics [6,7]. These studies demonstrated the successful encapsulation of various molecules within β-CD frameworks, improving stability and reducing the volatility of the active chemicals. The formation of inclusion complexes between β-CD and specific molecules through host–guest interactions enhances the encapsulated compounds’ stability and solubility [8]. β-CD microencapsulation has been also proposed to offer controlled and sustained release profiles of specific compounds. Overall, the use of β-CD in microencapsulation holds significant potential for a wide range of applications. It is crucial not only for extending the shelf life of volatile compounds and enhancing the efficacy of flavor agents, but also for increasing their antimicrobial and antioxidant properties, ensuring controlled or sustained release from the inclusion complex [9,10].

Allyl isothiocyanate (AITC), a compound known for its strong antimicrobial performance, is extensively used as a natural preservative in biomedical applications [11]. However, high volatility and rapid degradation limit its use in active packaging systems. By encapsulating AITC in β-CD, these limitations can be mitigated, enhancing the antimicrobial efficacy of the packaging material and changing the mechanical and thermal stability of the resulting active materials, making them effective for antimicrobial packaging [12].

Polylactic acid (PLA) possesses excellent properties such as bioabsorbability, biodegradability, mechanical strength, and transparency, which are essential for packaging applications [13]. The addition of active compounds to PLA matrices amplify its functionalities, offering effective solutions for reducing post-harvest losses and improving environmental sustainability [14]. Research has shown that incorporating β-CD complexes into PLA can influence the film’s physical properties, antimicrobial activity, and disintegration under composting conditions [15]. For instance, it has been demonstrated that β-CD inclusion complexes can improve the controlled release properties of active agents in biodegradable films, enhancing food safety and extending shelf life [16]. Shi et al. (2022) also observed an antimicrobial effect on releasing oregano essential oil from electrospun PLA-based nanofibers [17].

Previous studies explored the use of AITC-β-CD complexes in different materials, particularly in PLA films, for preservative purposes [18]. However, given the limited research until now on the compostability of these active films, this study aims to evaluate how the β-CD:AITC inclusion complexes affect the physical, chemical, and biological properties of PLA films when they disintegrate under composting conditions. This study also aims to determine the potential uses of these films as biodegradable antimicrobial material, while developing sustainable packaging solutions that meet environmental and functional standards. These findings have broad implications for the packaging industry, offering innovative approaches to managing waste and enhancing food preservation.

## 2. Results

### 2.1. Characterization of PLA Films

#### 2.1.1. Field Emission Scanning Electronic Microscopy (FESEM)

**Figure 1:**
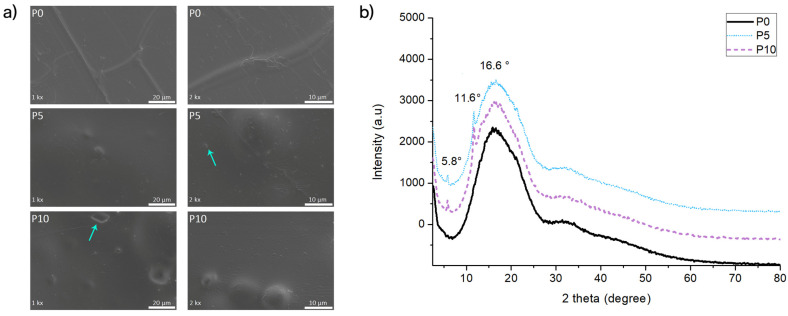
(**a**) FESEM micrographs; (**b**) XRD diffractograms of extruded PLA (P0) and active films (P5 and P10).

Figure 1a shows the surface morphological analysis of the PLA films with and without the addition of the inclusion complexes. P0 films showed a smooth surface, while active films exhibited structures with different morphologies on their surface. Indeed, structures with bubbles were evident on the surface of P5 and P10 films. Furthermore, small structures like flakes were also identified on these materials, as indicated with arrows in Figure 1. Previous studies on extruded films loaded with inclusion complexes also identified bubble-type structures in the material surface, which were associated with the agglomeration of fillers due to the low miscibility among all components in these blends [19,20,21,22]. However, the agglomeration is not apparent on the polymer surface, but it could be embedded in the matrix, producing some relief on the surface and some influence on mechanical and disintegration processes by accelerating the fragmentation or release of the active compound. This observation is related to the fact that the flake-like structures (pointed with arrows) on the surface of active films were similar to those observed in a previous study on the original inclusion complexes [23].

#### 2.1.2. X-Ray Diffraction (XRD)

The crystalline structure of films was studied using XRD analysis (Figure 1b). XRD patterns showed a broad band at 2θ = 16.6° for P0, which evidenced a polymer with an amorphous state [24,25]. On the other hand, for P5 and P10 films, the broad peak corresponding to PLA was observed along with characteristic crystallinity peaks at 11.6° and 5.8°, corresponding to the crystalline structure of inclusion complexes. This change in the polymer structure is related to the host–guest interaction, evidencing the AITC complexation [23]. This result evidenced the presence and distribution of the inclusion complexes into the polymeric matrix, which was also observed in the FESEM analysis. Therefore, it could be concluded that the extrusion process did not affect the original structure of inclusion complexes, which confirmed the thermal protection of the AITC by β-CD after the extrusion process.

#### 2.1.3. Thermal Properties

The thermal parameters obtained using TGA and DSC are summarized in Table 1. All films exhibited a one-step thermal degradation with maximum rate around 366 °C (T_deg_), indicating the occurrence of bond cleavage on the backbone to produce cyclic oligomers, lactide, and carbon monoxide as products [17]. It was evidenced that inclusion complexes were not able to modify the thermal stability of PLA.

On the other hand, the glass transition temperature (T_g_), cold crystallization temperature (T_cc_), melting temperature (T_m_), and enthalpies associated with the first-order transitions were obtained from DSC thermograms of different films. Results obtained for the P0 film were similar to those reported in other studies [26,27,28]. The presence of inclusion complexes did not modify the values of T_g_, but higher T_cc_ and T_m_ values were registered for P5 and P10. This fact was associated with the thermal stability of the inclusion complexes that withstand temperatures up to 300 °C [23], which could slightly improve the thermal behavior of the active films. Moreover, a decrease in cold crystallization and melting enthalpies (Δ*H_cc_* and Δ*H_m_*) in active films was observed. The reduction in Δ*H_cc_* could be related to a high free volume in the amorphous zone due to the presence of inclusion complexes, which generated higher chain mobility in the materials. Instead, the lower value of Δ*H_m_* was related to a decrease in crystallinity due to the low miscibility of all components in the inclusion complexes. Other authors have also reported similar results [19,26,29]. Finally, the PLA crystallinity was not modified, being lower than 5% for all samples, which agrees with the XRD results.

#### 2.1.4. Color Measurements

The visual aspect and the color parameters (L*, a*, b*, and the color difference ΔE) for all materials are shown in Table 1. A high value of L*, a value of a* close to 0, and a positive value of b* were found for P0, which means the film was luminous with a greenish tonality. Other researchers have also obtained similar color parameters for PLA films [19,28]. The incorporation of inclusion complexes β-CD:AITC into PLA affected significantly the color parameters of films. For instance, the highest concentration of complexes decreased L* and a* values, but b* increased to 2.57 ± 0.02 and 2.92 ± 0.09 for P5 and P10, respectively. Therefore, P5 and P10 materials were darker, with a higher yellow and green tone than P0. Nevertheless, active films showed ΔE value lower than 5. Likewise, perceptible color changes were not visible to the human eye [30].

#### 2.1.5. Mechanical Properties

The tensile parameters of films are shown in Table 1. The values of the elastic modulus, tensile strength, and elongation at the break of P0 were similar to PLA films reported in other works [26,31]. However, the incorporation of inclusion complexes significantly decreased all tensile parameters, obtaining more rigid and brittle films compared to P0. This behavior has also been reported in active PLA films loaded with inclusion complexes obtained from cyclodextrin and different active compounds [26,29,32]. In this study, the low compatibility between PLA and the inclusion complexes generated physical interactions that were able to limit the movement of the polymeric chains in the active films. Furthermore, the agglomeration of inclusion complexes in the polymeric matrix of the active films observed in Figure 1a could generate breakpoint zones in the materials that increased their fragility [28].

#### 2.1.6. Barrier Properties (Water Vapor and Oxygen Permeability)

Table 1 summarizes the OP and WVP of the films evaluated under different RH conditions. The permeability values of P0 were similar to those reported in previous works on PLA films [29,32]. Furthermore, the RH did not significantly influence the OP (*p*-value = 0.2101) and WVP (*p*-value = 0.6296) according to a multifactorial variance analysis. Auras et al. also reported a similar effect in PLA films from 40 to 90% RH. Regarding active films, the incorporation of inclusion complexes in P5 and P10 films did not produce significant changes in their OP [33].

However, the incorporation of inclusion complexes into P5 and P10 films produced a significant and systematic increase in the WVP of active films. This fact can be attributed to the presence of inclusion complexes on the surface of the films that favored the adsorption of water molecules. Furthermore, in a previous study, it was demonstrated that β-CD:AITC produced a more wettable film surface that could favor water diffusion [34]. This increase in WPV could be helpful for the packaging of fresh food to avoid their dehydration. In addition, active compound release could be favored; thus, antimicrobial protection for the product could be reached.

### 2.2. Releasing of AITC from Active Films (Responsive Capacity)

**Figure 2:**
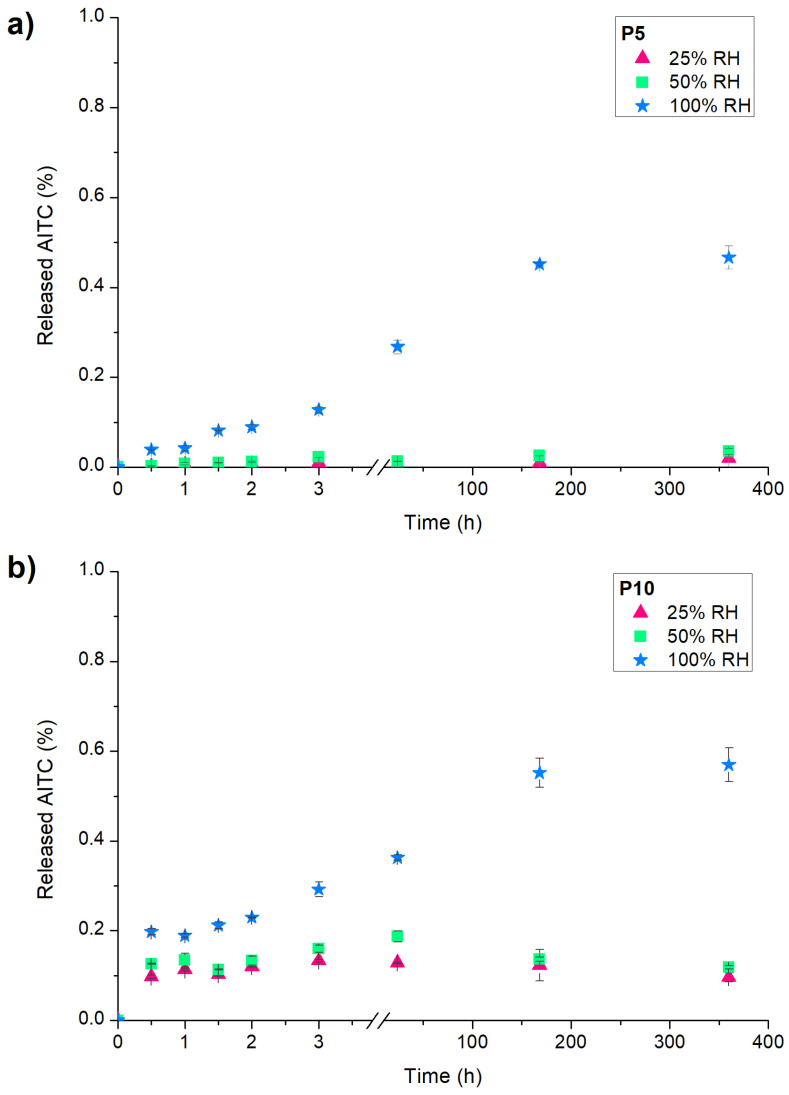
Release of AITC from active films to headspace at 20 °C and under different RHs (25, 50 and 100% RH): (**a**) P5; (**b**) P10 active films.

Figure 2a shows the curves of AITC release from active PLA films exposed to different RHs. A slow initial release was observed in all cases, and the plateau was reached after 166 h (7 days). However, the increase in RH produced a higher release of AITC from P5 and P10, demonstrating that these films are RH-responsive materials. This fact was attributed to the interaction of water molecules through hydrogen bonds with the inclusion complexes that trigger the AITC release [23]. This behavior shows that the materials could maintain their functionality at low RH values and release the active compound at high RH, which are important factors for considering the storage and application of these active films at an industrial scale as food packaging materials.

On the other hand, the percentage of released AITC from P5 and P10 was similar when they were exposed to the same RH. This result could be associated with a similar distribution of inclusion complexes in the polymeric matrix for both films, as the FESEM micrographs evidenced. Nevertheless, a higher AITC release percentage was expected from P10 since the agglomerations increased the diffusion rate of water molecules (according to barrier properties analysis), which could trigger the release of active compound from β-CD:AITC inclusion complexes [23]. Therefore, it seems that AITC was mainly released from inclusion complexes located on the surface of the active films instead of the embedded ones. This fact could be confirmed by the low percentage of AITC released, considering the low loss of active compound during extrusion since the structure of inclusion complexes were maintained in the process according to XRD and DSC analysis.

### 2.3. Disintegration Under Composting Conditions

The disintegration under composting conditions is a decomposition process of organic matter carried out by microorganisms to carbon dioxide, water, and heat. PLA-based materials are generally converted to small fragments during this natural process, and soil benefits for the plant growth are obtained [35]. The biodegradation of PLA starts with water diffusion through the polymeric matrix, producing a non-enzymatic hydrolysis of the polymer and a reduction in its molecular weight. Subsequently, oligomers and lactic acid obtained from the fragmentation of PLA are assimilated by microorganisms to be finally converted to carbon dioxide and water [36,37]. On the other hand, the biodegradation process of polymeric materials can be affected by the characteristics of the polymer, the environmental conditions, and the presence of additives. Therefore, the effect of incorporating β-CD:AITC inclusion complexes on the disintegration of PLA films under composting conditions was evaluated based on macroscopic changes (color, size, and texture); disintegration percentage; and morphological, structural, and thermal properties.

#### 2.3.1. Macroscopic Changes and Disintegration Percentage

**Figure 3:**
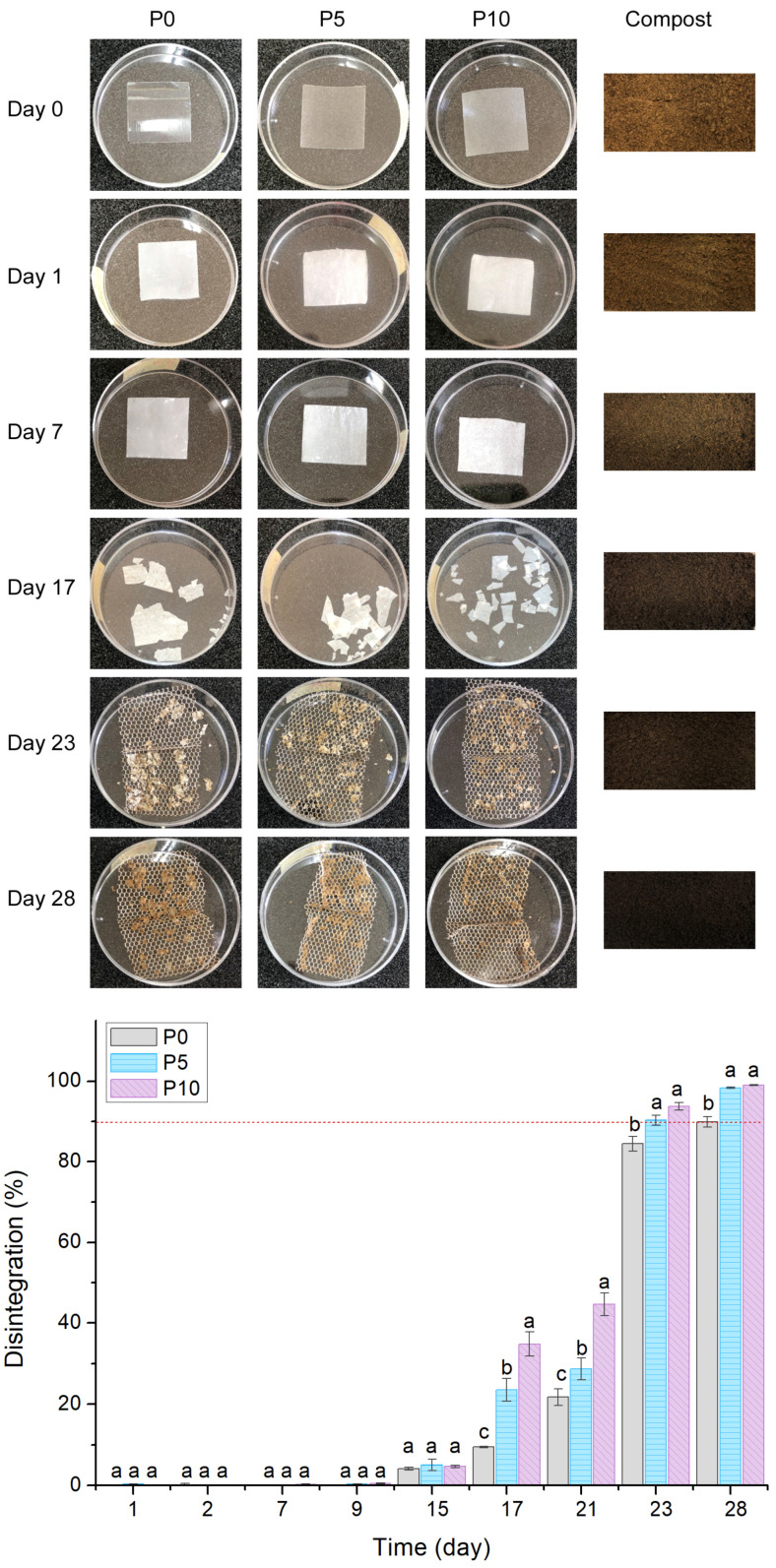
Disintegration of PLA and active films under composting conditions (dotted red line represent the 90% of the disintegration test established by the UNE-EN 13432 normative). Columns with the same letter (a, b, c) within a day show statistical similarity between films (*p* > 0.05) according to ANOVA and Tukey tests.

Figure 3 shows the disintegration percentage of PLA films and the changes that occurred in their visual appearance. The whitening of films produced an increase in their opacity at day 1. These visual changes have been widely reported as the result of the hydrolytic degradation of PLA due to the water intake from compost to the amorphous sites of the polymer matrix [38]. Furthermore, the disintegration percentage of films was not modified up to day 9. This fact is associated with the change in the polymer crystallinity and its method of initial disintegration (surface erosion), and this will be further discussed [39]. At day 15, all films exhibited a disintegration percentage of 5%, similar to results reported in other studies [36,40]. At day 17, the disintegration percentage of all materials started to increase, and samples lost their physical integrity, as evidenced by the fragmented films’ appearance. This phenomenon occurred due to the beginning of the polymer hydrolysis and the consequent assimilation of the short polymer chains by microorganisms.

Furthermore, it was evidenced that the highest content of inclusion complexes favored the disintegration percentage and fragility of films. These changes are associated with the mechanical and barrier characteristics of active films. The lower tensile strength obtained for P5 and P10 films was related to the agglomeration of inclusion complexes, which served as breakpoints, to improve the fragmentation of active films. Moreover, inclusion complexes increased the WVP of the polymer matrix due to their hydrophilic nature. As a result, active films could absorb and diffuse higher amounts of water from compost, producing a higher hydrolysis of PLA. On the other hand, the rupture of polymer chains possibly exposed the β-CD of inclusion complexes to microorganisms that could assimilate it as a substrate. This fact could support the results obtained on day 23, where both active films reached the objective of the disintegration test established by the UNE-EN 13432 normative (represented by the dotted red line), while P0 reached 90% disintegration on day 28 [41].

Regarding the physicochemical properties of synthetic solid bio-waste (compost), it started with pH = 5.4, volatile solids = 17.7% and dry solids = 48.0%, which indirectly showed around 50% moisture as the ISO 20200:2023 standard requires [42]. After 28 days, the pH increased to 8.0, while volatile and dry solids decreased to 10.7% and 28.4%, respectively. This means that the compost showed an alkaline pH, higher carbon amounts, and around 72% moisture. These physicochemical variations, as well as the color change in the compost, evidenced the generation of water, ammonium, nitrogen, carbon, and other substances derived from aerobic fermentation, which in turn confirmed the composting trial success during PLA and active film disintegration.

#### 2.3.2. Morphological Analysis

**Figure 4:**
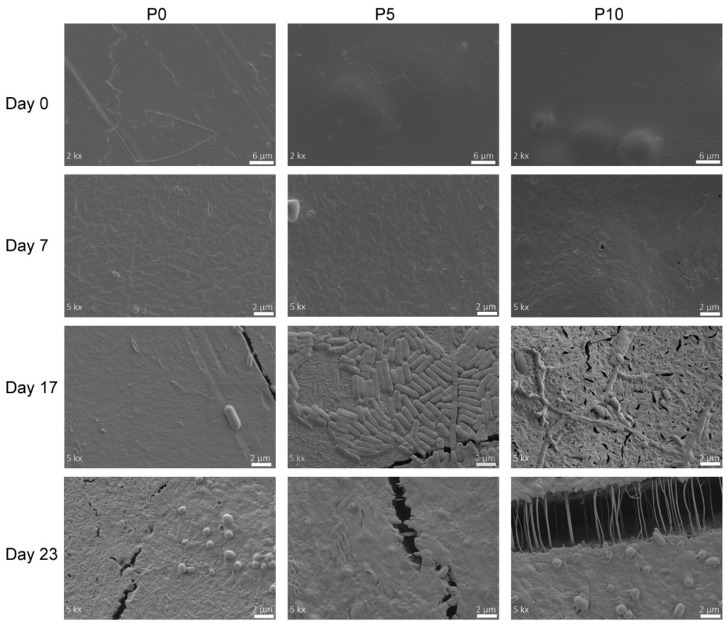
FESEM surface micrographs of the films subjected to the disintegration process under composting conditions.

The changes occurring on the surface of the PLA films during their disintegration were followed using FESEM. Figure 4 shows the micrographs of the materials subjected to different composting times. All films exhibited smooth surfaces and a highly compact structure during the first days of the trial. Furthermore, the presence of inclusion complexes in P5 and P10 films was also observed. On day 7, an increase in surface roughness was observed, which was associated with the beginning of film erosion caused by changes in crystallinity. In addition, the inclusion complexes contained in active films were exposed to the surface, and in the P10 film, the presence of small holes was evidenced. On day 17, some fractures in the materials surface were observed, in agreement with the increase in fragility caused by the disintegration process. For instance, the P0 film showed some fractures although its surface was still mainly even. Instead, P5 and P10 films exhibited highly fractured surfaces due to the remarkable hydrolytic degradation favored by the high water intake into the polymer matrix. Furthermore, micrographs of both active films showed that small polymeric chains produced by the disintegration started to be assimilated by the microorganisms, whose morphology is similar to *Stenotrophomonas pavanii*, a PLA-degrading bacteria [43]. It is important to note that the incorporation of inclusion complexes might delay the disintegration process due to the antimicrobial activity of AITC. However, the development of microorganisms was not significantly affected because of the low concentration of the active compound. Moreover, it was observed in the WVP analysis that inclusion complexes enhanced the diffusion of water through the matrix, improving PLA hydrolysis [44]. The advancement of the disintegration process (23 days in Figure 4) favored higher damage on the surface of P5 and P10 films. Thus, larger fractures were generated as the result of the strong hydrolysis of PLA. Furthermore, as this hydrolysis mainly occurred in amorphous zones, small crystalline spherulites appeared on the films surface, corresponding to crystalline sites of PLA that are further degraded [36].

#### 2.3.3. FTIR Analysis

**Figure 5:**
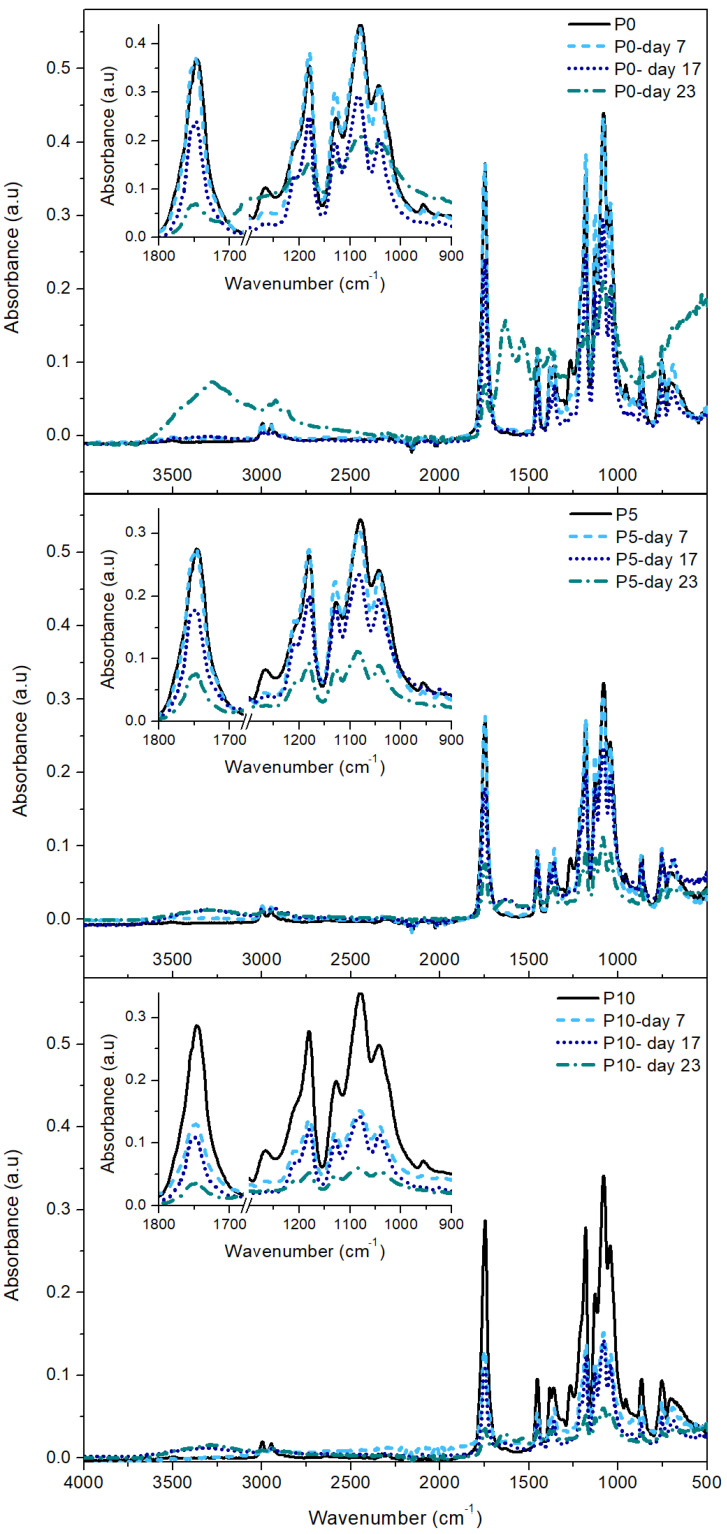
FTIR spectra of the films subjected to the disintegration process under composting conditions.

Changes in the chemical structure of PLA and active films can be determined through the analysis of FTIR spectra, shown in Figure 5. The FTIR analysis focused on the characteristic bands of PLA [27] associated with some specific regions of the spectra: (I) crystalline zone (920 and 1210 cm^−1^); (II) amorphous zone (954 and 1268 cm^−1^); (III) C-O and C-C-O bonds (bands from 1000 to 1150 cm^−1^); (IV) stretching of the carbonyl group C=O (1744 cm^−1^); and (V) C-H bonds of the polymeric chain (bands from 2900 to 3000 cm^−1^).

Since all films were amorphous at day 0, the bands related to the crystalline zone of PLA were not detected in the corresponding spectrum. On day 7, a decrease in intensity of the bands at 954 and 1268 cm^−1^ and the appearance of two new bands at 920 and 1210 cm^−1^ were observed. These changes are related to the increase in the crystalline domains of the samples with low molecular weight as a result of the beginning of hydrolysis in the more accessible amorphous zones [39]. This fact, in turn, supports the changes observed in the visual appearance of the films during the first stage of the degradation trial. At day 17, the intensity of the bands associated with the carbonyl group and with C-O and C-C-O bonds decreased. These changes confirmed the hydrolytic scission of ester groups of PLA and the formation of lactic acid, lactide, and oligomers [45]. The presence of two new bands in P5 and P10 films was remarkable. One band was located at 3300 cm^−1^ and associated with the presence of water in the polymer matrix and the exposition of hydroxyl groups of β-CD toward the film surface, while a small band detected at 1630 cm^−1^ was related with the presence of free carboxylate ions produced by the assimilation of lactic acid and oligomers by microorganisms. These findings support the fact that inclusion complexes facilitated the intake of water molecules into the polymer matrix, accelerating the hydrolytic degradation of PLA and the appearance of fractures in the materials, as observed by FESEM. These results demonstrated the increase in material fragility due to the effect of the disintegration process of the polymer matrix. On day 23, a notable decrease in the intensity of all FTIR bands was observed in all materials, especially in active films. Furthermore, the bands between 2900 and 3000 cm^−1^ disappeared, showing the collapse of the polymeric structure by the depletion of lactic acid and the final stretch of the disintegration process observed after 28 days [46].

#### 2.3.4. Thermal Analysis (TGA and DSC)

**Figure 6:**
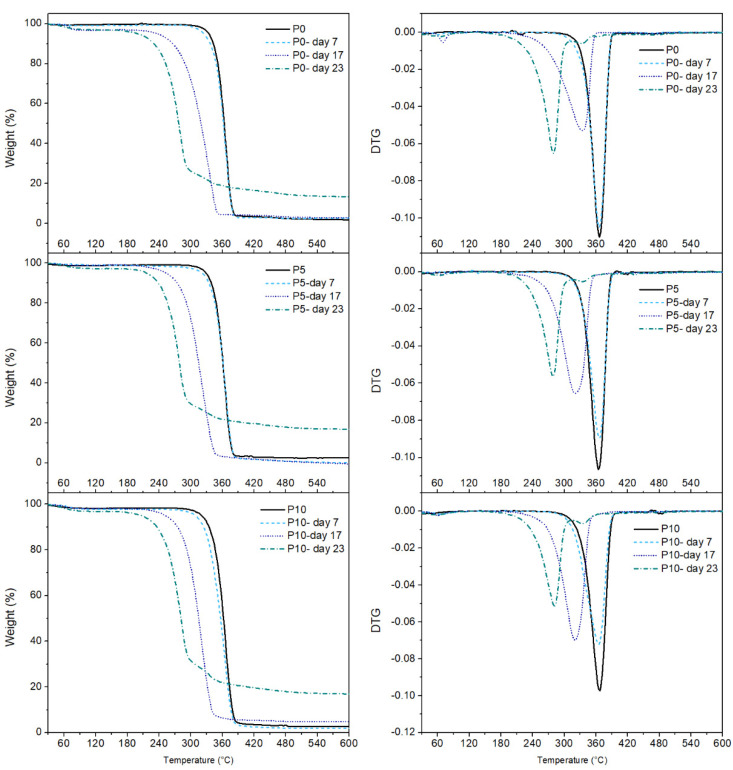
TGA and DTGA curves of the films subjected to the disintegration process under composting conditions.

Figure 6 shows the TGA and DTGA curves of the films, while the main data are summarized in Table 2. All samples exhibited only one thermal degradation process related to PLA. However, disintegration affected the samples’ maximum degradation temperature (T_deg_). At day 0, all samples showed a maximum weight loss of films around 366 °C, which was maintained at day 7. This result confirmed that surface erosion occurred during this time, as previously discussed. The significant decrease in T_deg_ for all films at day 17 was associated with the reduction in the polymer molecular weight caused by the advanced hydrolysis process and the consequent microbial attack [45]. However, this phenomenon primarily affected P5 and P10 films since their T_deg_ was lower than the T_deg_ for the P0 film, confirming that the presence of inclusion complexes improved polymer hydrolysis, generating shorter polymeric chains, which need a lower temperature for degradation. At day 23, all materials exhibited a reduction of ≈70 °C from the initial T_deg_, showing the loss of the polymeric structure caused by degradation.

**Figure 7:**
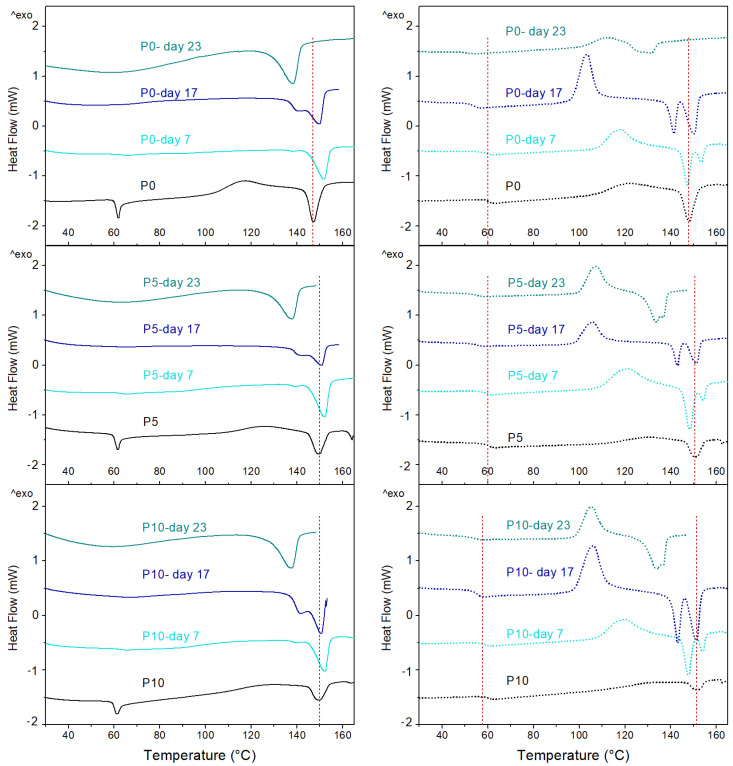
DSC thermograms obtained during the first heating (**left**) and second heating (**right**) of the PLA (P0) and active (P5 and P10) films.

On the other hand, DSC thermograms obtained during the first and second heating scans are shown in Figure 7. Furthermore, the DSC parameters obtained from different thermal events are detailed in Tables S1 and S2. At day 0, the glass transition temperature (T_g_) of PLA in P0, P5, and P10 was observed at 60 °C, while an endothermic peak characteristic of the enthalpic relaxation phenomenon was also observed [47], followed by the cold crystallization (T_cc_) around 120 °C and melting (T_m_) at 150 °C. During the second heating scan, the peak associated with the enthalpy relaxation of PLA disappeared, and cold crystallization and melting enthalpies (Δ*H_cc_* and Δ*H_m_*) were reduced. These changes were associated with the slow reorganization of polymer chains during the cooling step.

On day 7, peaks corresponding to the enthalpy relaxation and cold crystallization of PLA disappeared in all films, while values for the T_m_, Δ*H_m_*, and crystallinity (*X_c_*) of materials were increased. These results are related to the hydrolysis of the amorphous zones of PLA since they are responsible for these thermal transitions. For instance, the polymer amorphous zones are responsible for the cold crystallization [38,39,45]. Moreover, the polymer amorphous zones increase the enthalpy relaxation, and a reduction in these zones decreases relaxation [47]. Additionally, the decrease in the amorphous zones produces an increase in the polymer’s overall crystallinity, resulting in the need for higher melting energy. From day 17, T_g_ was not visible for any sample, and a small shoulder before the polymer melting was detected. This effect is attributed to the break in the polymer chains and the microbial attack to yield oligomers with different molecular weights and the formation of different crystalline structures able to melt at different temperatures [45,46]. Furthermore, the T_m_ of active films was reduced compared to values registered at day 7. This change supports the observation of a higher hydrolytic degradation of the active films. At day 23, a significant decrease in the melting temperature of materials was observed due to the high disintegration of samples, which triggered very small polymer chains. Furthermore, the polymer’s crystallinity was significantly increased, which supports the observations in the FESEM analysis, where crystalline spherulites were detected on the film surfaces.

On the other hand, the DSC thermograms obtained during the second heating of the disintegrated films (from day 7) also showed the thermal events associated with glass transition, cold crystallization, and the double melting peak of PLA. The progress in the disintegration process of materials progressively decreased the T_g_ value of the polymer, while Δ*H_cc_* and Δ*H_m_* increased. These findings were attributed to changes in the amorphous/crystalline ratio of the polymeric matrix by the effect of the PLA hydrolysis. The presence of shorter polymeric chains improved the molecular mobility resulting in a lower T_g_. The high molecular mobility caused a higher ordering of amorphous zones of the polymer and their later crystallization during heating. This fact is evident since the DSC thermograms of the second heating of the films showed polymer cold crystallization, unlike the first heating. Furthermore, the presence of the two melting peaks in the films was associated with the different ways of PLA crystallization due to the heterogeneity in the molecular size of the polymer chains [36,46]. These observations agreed with the presence of spherulites on the surface of the films as observed in the FESEM micrographs at day 17.

## 3. Materials and Methods

### 3.1. Materials

β-Cyclodextrin (purity > 98.5%) was obtained from Cyclolab, Ltd. (Budapest, Hungary). The active compound allyl isothiocyanate (AITC > 95%) was purchased from Sigma Aldrich (Santiago, Chile). Poly(lactic acid) resin (PLA) 2003D Ingeo™ was obtained from NatureWorks (Minnetonka, MN, USA). β-CD:AITC inclusion complexes (1:1 molar ratio) were obtained using a co-precipitation method reported in a previous study [23].

### 3.2. Film Preparation

PLA powder was dried at 50 °C for 48 h. Dried PLA or its different mixtures with inclusion complexes were melt-extruded in a 20 mm co-rotating laboratory twin-screw extruder Labtech LTE20 (Praksa, Thailand). A temperature profile from 190 °C to 210 °C (feed zone to die zone) was used for the extrusion, and films were collected in an attached chill roll (Samutprakarn, Thailand) at 2 m min^−1^. Films were denoted as P0, P5, and P10 for neat PLA, PLA with 5% wt, and 10% wt of the inclusion complex, respectively. Concentrations were chosen based on the release behavior and antifungal activity of inclusion complexes reported in a previous study [23].

### 3.3. Film Characterization

#### 3.3.1. Field Emission Scanning Electronic Microscopy (FESEM)

Micrographs of the materials surface were taken in 1 kx and 2 kx using a FESEM Supra 25-Zeiss microscopy (Jena, Germany). Prior to FESEM measurements, samples were coated with a tungsten layer to enhance their electrical conductivity.

#### 3.3.2. X-Ray Diffraction (XRD)

XRD diffractograms of samples were recorded between 2° and 80° of the 2θ angle at 0.05 Hz, 40 mA, 40 kV, and CuKα radiation (k = 1.54 Å) using a Bruker D8-Advance diffractometer (Madison, WI, USA).

#### 3.3.3. Thermogravimetric Analysis (TGA)

Samples of 5–7 mg were put in an alumina crucible, and they were heated from 25 °C to 700 °C at 10 °C min^−1^ in a nitrogen atmosphere (50 mL min^−1^) using a TGA/SDTA 851e equipment (Mettler Toledo, Schwarzenbach, Switzerland). The maximum degradation temperature (T_deg_) of each sample was registered. The analysis was done in triplicate.

#### 3.3.4. Differential Scanning Calorimetry (DSC)

Samples of 3 mg (in triplicate) were introduced in a sealed aluminum crucible and submitted to a first heating from 0 to 160 °C, a cooling from 160 °C to 0 °C, and a second heating from 0 °C to 150 °C. All scans were performed at 10 °C min^−1^ in a nitrogen atmosphere (50 mL min^−1^) using a DSC Q2000 (TA Instruments, New Castle, DE, USA). The percentage of crystallinity (*X_c_*) in the samples was calculated from DSC thermograms using Equation (1):

(1) $$X_{c}\;\left(\%\right)=\frac{\Delta H_{m}-\Delta H_{cc}}{\Delta H_{m}^{0}\;\left(1-X_{IC}\right)}\times 100$$

where Δ*H_m_* and Δ*H_cc_* are the melting and cold crystallization enthalpies associated with T_m_ (melting temperature) and T_cc_ (cold crystallization temperature), respectively; Δ*H_m_*^0^ was the enthalpy for 100% crystalline PLA (93 J g^−1^ according to Xu et al. (2017) [48]); and *X_IC_* was the fraction of inclusion complexes incorporated to the film (0.05 for P5 and 0.10 for P10).

#### 3.3.5. Color Measurements

CIEL*a*b* parameters of the materials were obtained using a CR-410 Minolta Chroma Meter colorimeter (Minolta Series, Tokyo, Japan) with a D65 illuminant, 2° observer, and a standard white background (L* = 97.76, a* = −0.03 and b* = 1.87). The reported values were the mean of ten measurements for each film.

#### 3.3.6. Mechanical Properties

The tensile properties were determined using INSTRON 3344 equipment (Illinois Tool Works, Glenview, IL, USA) with a charge of 2 kN, initial separation of 125 mm, and speed of 12.5 mm min^−1^, according to the ASTM D882-09 norm [49]. The reported values were the mean of ten measurements for each film probe (150 mm × 2.5 mm).

#### 3.3.7. Barrier Properties

Barrier properties considered as oxygen permeability (OP) and water vapor permeability (WVP) were evaluated at 50 and 80% RH, respectively. The OP was determined at 23 °C using OXTRAN (MOCON, Brooklyn Park, MN, USA) equipment according to the ASTM D3985-17 standard [50]. The WVP values of films were determined using a Systech Illinois M7002 water permeation analyzer (Industrial Physics, New Castle, DE, USA) at 37.8 °C, according to ASTM F1249-13 standard [51].

### 3.4. Release Vapor Phase (Responsive Capacity)

The AITC release kinetics from the materials to headspace was determined at 25, 50, and 100% RH. Saturated salt solutions (0.6 mL) of potassium acetate, magnesium nitrate, or distilled water were used inside 22 mL chromatograph vials to keep RH values. A film sample of 4 × 5 cm^2^ was rolled up in the top of the vial to avoid contact with the salt solution, and it was sealed with an aluminum lid coupled to a silicone/PTFE septum. Vials were stored at 20 °C and analyzed at different times using a Turbomatrix 40 headspace analyzer coupled to a Clarus 580 gas chromatograph (Perkin Elmer, Shelton, CT, USA). Three vials for each sample were prepared. For AITC quantification, the headspace autosampler took 0.2 mL of the headspace from the vial and transferred it to the gas chromatograph (GC). The injection temperature was set at 240 °C. The column temperature started at 40 °C for 0.5 min, and then it was heated to 120 °C at 20 °C min^−1^. This temperature was maintained for 0.5 min. Finally, it increased to 270 °C at the same heating rate, keeping this temperature for 0.5 min. Helium was used as the carrier gas at 11 psi, and the flame ionization detector (FID) was programmed at 260 °C. The released AITC at each time point was quantified in triplicate using a calibration curve previously prepared with standards from 0 to 0.3 mg AITC L_air_ (R^2^ = 0.9970).

### 3.5. Disintegration Under Compost Conditions

The disintegration trial was performed at a laboratory scale according to the ISO 20200:2015 standard. Films were cut into 25 × 25 mm^2^ pieces, weighted, and placed in a textile mesh to facilitate the removal during composting test. Samples were buried at approximately 6 cm depth in perforated plastic boxes with a moist synthetic solid bio-waste (40% of sawdust, 30% of rabbit food, 10% starch, 10% compost, 5% sugar, 3% corn oil, and 2% urea with 50 wt% of water). These reactors were stored at 58 °C and 50% RH in aerobic conditions. Bio-waste was scrambled, and evaporated water was restored according to the norm. Samples were analyzed at different times (0, 1, 2, 7, 9, 15, 17, 21, 23, and 28 days), by removing them from the textile mesh, washing with deionized water, drying for 24 h, and weighing. The percentage of disintegration at each time was calculated using Equation (2):

(2) $$Disintegration\;\left(\%\right)=\frac{W_{i}-W_{t}}{W_{i}}\times 100$$

where *W_i_* and *W_t_* correspond to the sample weight before the assay and after the removal and drying operations, respectively.

Photographs of test samples were taken when a principal visual change occurred (7, 17, and 23 days). In addition, these samples were analyzed using FESEM, TGA, DSC, and Fourier transform infrared spectroscopy (FTIR) to follow the morphological, thermal, and chemical changes of films during the disintegration process. The physicochemical properties of synthetic solid bio-waste, such as pH, volatile and dry solids, and color, were also assessed before and after the trial.

## 4. Conclusions

The incorporation of β-CD:AITC inclusion complexes into PLA matrices using extrusion processing results in major changes in the morphological, thermal, and barrier properties of the obtained films. Although inclusion complexes were agglomerated in the polymer matrix, they improve the thermal stability of the polymer. Moreover, the oxygen permeability of PLA was not modified by the addition of inclusion complexes, but they produced higher water vapor permeability due to their high water-sorption capacity. In addition, non-detectable color changes were produced by the incorporation of inclusion complexes in PLA, which is an important parameter for food packaging applications. Furthermore, the incorporation of inclusion complexes in PLA films reduced the disintegration time of materials under composting conditions by 5 days, achieving the maximum disintegration rate after 23 days. The water absorption capacity of the inclusion complexes is the main reason for this reduction by accelerating polymer hydrolysis. This fact was supported by the significant changes in the morphological, chemical, structural, and thermal properties of the active films during the disintegration trial.

The release of AITC in the headspace was promoted by the increase in RH, regardless of the inclusion complex concentration. This means that PLA/inclusion complex films are RH-responsive materials that can maintain their functionality at low RH and release the active compound within a specific time under controlled conditions. This behavior could be useful for determining optimal material storage conditions and for applications in foods that require antimicrobial properties at high moisture, such as fruit and vegetables.

## Figures and Tables

**Table 1:** Physicochemical properties of PLA (P0) and active films (P5 and P10). Means with the same letter within a row shows statistical similarity between the samples (*p* > 0.05) according to the ANOVA and Tukey tests.

		P0	P5	P10
		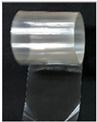	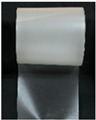	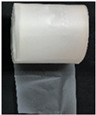
**Thermal events**				
Maximum degradation temperature/T_deg_ (°C)		367.0 ± 0.5 ^a^	366.1 ± 1.0 ^a^	366.1 ± 0.7 ^a^
Glass transition temperature/T_g_ (°C)		60.4 ± 0.7 ^a^	60.7 ± 0.1 ^a^	60.6 ± 0.2 ^a^
Cold crystallization temperature/T_cc_ (°C)		120.9 ± 0.1 ^b^	130.0 ± 0.5 ^a^	131.2 ± 0.4 ^a^
Cold crystallization enthalpy/Δ*H_cc_* (J g^−1^)		16.8 ± 0.2 ^a^	6.6 ± 0.2 ^b^	2.7 ± 0.2 ^c^
Melting temperature/T_m_ (°C)		148.0 ± 0.2 ^b^	151.1 ± 0.1 ^a^	151.8 ± 0.3 ^a^
Melting enthalpy/ΔH_f_ (J g^−1^)		19.0 ± 0.5 ^a^	10.6 ± 0.4 ^b^	6.0 ± 0.5 ^c^
Crystallinity/X_c_ (%)		2.3 ± 0.8 ^a^	4.3 ± 0.6 ^a^	3.5 ± 0.8 ^a^
**Color parameters**				
L*		98.23 ± 0.09 ^a^	98.01 ± 0.06 ^b^	97.25 ± 0.11 ^c^
a*		−0.06 ± 0.01 ^a^	−0.09 ± 0.01 ^b^	−0.12 ± 0.01 ^c^
b*		2.28 ± 0.04 ^c^	2.57 ± 0.02 ^b^	2.92 ± 0.09 ^a^
ΔE		-	0.36 ± 0.05 ^b^	1.18 ± 0.14 ^a^
**Mechanical properties**				
Young’s modulus (MPa)		3031.3 ± 148.3 ^a^	2142.2 ± 196.0 ^b^	2148.8 ± 134.0 ^b^
Tensile strength (MPa)		87.4 ± 2.8 ^a^	75.1 ± 5.9 ^b^	73.7 ± 4.1 ^b^
Elongation at break (%)		1.96 ± 0.06 ^a^	1.82 ± 0.12 ^ab^	1.73 ± 0.06 ^b^
**Barrier properties**				
OP × 10^−6^ (cc·m/m^2^·d·Pa)	50 %HR	1.6 ± 0.1 ^a^	1.6 ± 0.1 ^a^	1.5 ± 0.1 ^a^
	80 %HR	1.4 ± 0.1 ^a^	1.2 ± 0.1 ^a^	1.7 ± 0.2 ^a^
WVP × 10^−7^ (g·m/m^2^·d·Pa)	50 %HR	9.7 ± 0.1 ^c^	11.8 ± 0.3 ^b^	17.3 ± 0.2 ^a^
	80 %HR	9.9 ± 0.2 ^c^	12.5 ± 0.4 ^b^	16.8 ± 0.1 ^a^

**Table 2:** Results of TGA analysis of PLA (P0) and active (P5 and P10) films subjected to the disintegration process under composting conditions.

	T_deg_ (°C)		
	P0	P5	P10
**Day 0**	367.03 ± 0.50 ^aA^	366.19 ± 1.02 ^aA^	366.09 ± 0.66 ^aA^
**Day 7**	366.38 ± 0.05 ^aA^	366.29 ± 0.07 ^aA^	365.54 ± 0.71 ^aA^
**Day 17**	333.35 ± 1.24 ^aB^	322.83 ± 2.90 ^bB^	319.08 ± 1.38 ^bB^
**Day 23**	280.54 ± 1.79 ^aC^	278.16 ± 0.51 ^aC^	280.67 ± 0.98 ^aC^

Means with the same lower-case letters (a, b) within a row show statistical similarity among the films (*p* > 0.05) according to ANOVA and Tukey tests. Means with the same capital letters (A, B, C) within a column show statistical similarity among the days (*p* > 0.05) according to ANOVA and Tukey tests.

## Data Availability

Data are contained within the article and Supplementary Materials.

## Supplementary Materials

The following supporting information can be downloaded at: https://www.mdpi.com/article/10.3390/molecules29245859/s1, Table S1: DSC parameters obtained during the first heating of the PLA (P0) and active (P5 and P10) films subjected to the disintegration process under composting conditions; Table S2: DSC parameters obtained during the second heating of the PLA (P0) and active (P5 and P10) films subjected to the disintegration process under composting conditions.
