# Supplementary material for: Release and Disintegration Properties of Poly(lactic Acid) Films with Allyl Isothiocyanate-β-Cyclodextrin Inclusion Complexes for Active Food Packaging
Source: Molecules. 2024 Dec 12;29(24):5859. doi: 10.3390/molecules29245859 (PMC11677350; doi:10.3390/molecules29245859)
Supplement: Supplementary file 1 [file molecules-29-05859-s001.zip › molecules-3330888-supplementary.pdf]

SUPPLEMENTARY MATERIAL

Release and Disintegration Properties of Poly(lactic Acid) Films with Allyl Isothiocyanate-β-Cyclodextrin Inclusion Complexes for Active Food Packaging

Cristina Muñoz-Shugulí[1,2], Francisco Rodríguez Mercado[2], Abel Guarda[2], María José Galotto[2], Alfonso Jiménez[3], María Carmen. Garrigós[3], Marina Ramos[3*]

1 Facultad de Ciencias, Escuela Superior Politécnica de Chimborazo (ESPOCH), Riobamba EC060155, Ecuador; cristina.munoz@espoch.edu.ec
2 Packaging Innovation Center (LABEN-Chile), University of Santiago of Chile (USACH), Santiago 9170124, Chile; francisco.rodriguez.m@usach.cl (F.R.-M.); abel.guarda@usach.cl (A.G.); maria.galotto@usach.cl (M.J.G.)
3 Department of Analytical Chemistry, Nutrition & Food Sciences, University of Alicante, 03690 Alicante, Spain; alfjimenez@ua.es (A.J.); mc.garrigos@ua.es (M.C.G.)

Table S1. DSC parameters obtained during the first heating of the PLA (P0) and active (P5 and P10) films subjected to the disintegration process under composting conditions. Means with the same lower-case letters (a, b) within a row show statistical similarity between the films ($p > 0.05$) according to ANOVA and Tukey tests. Means with the same capital letters (A, B, C) within a column show statistical similarity between the days ($p > 0.05$) according to ANOVA and Tukey tests.

Parameter		P0	P5	P10
T_g (°C)	Day 0	61.1 ± 0.1^{bA}	60.3 ± 0.1^{bA}	60.2 ± 0.5^{bA}
	Day 7	63.3 ± 0.6^{aA}	62.5 ± 0.5^{aA}	63.0 ± 0.1^{aA}
	Day 17	-	-	-
	Day 23	-	-	-
T_{cf} (°C)	Day 0	116.3 ± 0.1^{B}	125.2 ± 0.4^{A}	124.2 ± 0.2^{A}
	Day 7	-	-	-
	Day 17	-	-	-
	Day 23	-	-	-
ΔH_{cf} (J g^{-1})	Day 0	18.2 ± 0.5^{A}	13.8 ± 1.0^{B}	8.9 ± 0.6^{C}
	Day 7	-	-	-
	Day 17	-	-	-
	Day 23	-	-	-
T_f (°C)	Day 0	147.3 ± 0.1^{cB}	149.6 ± 0.1^{bA}	149.8 ± 0.1^{bA}
	Day 7	152.0 ± 0.1^{aA}	152.3 ± 0.1^{aA}	152.9 ± 0.9^{aA}
	Day 17	149.8 ± 0.2^{bA}	150.1 ± 0.6^{bA}	150.7 ± 0.3^{bA}
	Day 23	138.6 ± 0.1^{dA}	138.2 ± 0.3^{cA}	137.8 ± 0.1^{cA}
ΔH_f (J g^{-1})	Day 0	19.1 ± 0.3^{dA}	15.5 ± 0.6^{dB}	12.6 ± 0.7cC
	Day 7	20.7 ± 0.4^{cB}	23.3 ± 0.7^{cA}	21.7 ± 0.3^{bAB}
	Day 17	35.5 ± 0.5^{aA}	28.6 ± 1.9^{bB}	23.5 ± 0.6^{bC}
	Day 23	33.1 ± 0.5^{bB}	36.1 ± 0.9^{aA}	32.2 ± 0.1^{aB}
X_c (%)	Day 0	1.0 ± 0.2^{dB}	1.9 ± 0.5^{dB}	4.0 ± 0.2^{dA}
	Day 7	22.3 ± 0.4^{cB}	25.0 ± 0.8^{cA}	23.3 ± 0.3^{cAB}
	Day 17	38.2 ± 0.5^{aA}	30.7 ± 2.1^{bB}	25.2 ± 0.7^{bC}
	Day 23	35.6 ± 0.5^{bB}	38.9 ± 0.9^{aA}	34.7 ± 0.2^{aB}

Table S2. DSC parameters obtained during the second heating of the PLA (P0) and active (P5 and P10) films subjected to the disintegration process under composting conditions. Means with the same lower-case letters (a, b) within a row show statistical similarity between the films ($p > 0.05$) according to ANOVA and Tukey tests. Means with the same capital letters (A, B, C) within a column show statistical similarity between the days ($p > 0.05$) according to ANOVA and Tukey tests.

Parameter		P0	P5	P10
**T_g (°C)**	Day 0	60.4 ± 0.7^{aA}	60.7 ± 0.1^{aA}	60.6 ± 0.2^{aA}
	Day 7	58.5 ± 0.6^{aA}	58.8 ± 0.6^{aA}	58.8 ± 0.4^{aA}
	Day 17	53.3 ± 0.1^{bA}	54.5 ± 0.1^{bA}	55.2 ± 1.0^{bA}
	Day 23	50.0 ± 0.9^{cB}	54.7 ± 0.8^{bA}	54.5 ± 0.7^{bA}
**T_{cf} (°C)**	Day 0	120.9 ± 0.1^{aB}	130.0 ± 0.5^{aA}	131.2 ± 0.4^{aA}
	Day 7	117.5 ± 0.9^{aB}	120.7 ± 0.5^{bA}	118.9 ± 0.9^{bAB}
	Day 17	103.4 ± 0.1^{bB}	104.4 ± 1.2^{dAB}	106.6 ± 0.5^{cA}
	Day 23	110.5 ± 1.5^{cA}	111.6 ± 1.4^{cA}	105.6 ± 0.1^{cB}
**ΔH_{cf} (J g^{-1})**	Day 0	16.8 ± 0.2^{cA}	6.6 ± 0.2^{bB}	2.7 ± 0.2^{dC}
	Day 7	23.1 ± 0.7^{bB}	28.4 ± 0.9^{aA}	22.6 ± 1.5^{cB}
	Day 17	35.9 ± 0.4^{aA}	29.4 ± 2.8^{aA}	34.1 ± 1.0^{aA}
	Day 23	11.5 ± 2.1^{dB}	29.5 ± 1.3^{aA}	27.3 ± 0.2^{bA}
**T_f (°C)**	Day 0	148.0 ± 0.2^{bB}	151.1 ± 0.1^{aA}	151.8 ± 0.3^{aA}
	Day 7	146.9 ± 0.4^{bB}	148.2 ± 0.2^{aA}	147.7 ± 0.1^{bAB}
	Day 17	149.9 ± 0.5^{aA}	140.5 ± 2.7^{bB}	143.3 ± 0.4^{cAB}
	Day 23	131.7 ± 0.2^{cC}	135.2 ± 0.6^{bA}	133.6 ± 0.2^{dB}
**ΔH_f (J g^{-1})**	Day 0	19.0 ± 0.5^{cA}	10.6 ± 0.4^{cB}	6.0 ± 0.5^{dC}
	Day 7	24.4 ± 0.5^{bB}	30.9 ± 0.5^{bA}	25.0 ± 1.2^{cB}
	Day 17	44.6 ± 1.0^{aA}	37.4 ± 1.5^{aB}	41.8 ± 1.0^{aAB}
	Day 23	14.2 ± 1.4^{dB}	30.8 ± 1.1^{bA}	29.7 ± 0.3^{bA}
**X_c (%)**	Day 0	2.3 ± 0.8^{bA}	4.3 ± 0.6^{bA}	3.5 ± 0.8^{bA}
	Day 7	1.5 ± 0.2^{bA}	2.7 ± 0.4^{bA}	2.6 ± 0.2^{bA}
	Day 17	9.3 ± 0.6^{aA}	8.6 ± 1.4^{aA}	8.3 ± 0.1^{aA}
	Day 23	2.9 ± 0.7^{bA}	1.4 ± 0.2^{bA}	2.5 ± 0.6^{bA}
